# Preoperative Diagnosis of Idiopathic Myointimal Hyperplasia of Mesenteric Veins in a Post–Liver Transplant Patient

**DOI:** 10.1016/j.gastha.2026.100965

**Published:** 2026-04-11

**Authors:** Ryan F. Hughes, Danielle Morelli, Eliza Cricco-Lizza, Emily Schonfeld, Arun B. Jesudian, Jose Jessurun, Carl V. Crawford

**Affiliations:** 1 Department of Medicine, NewYork-Presbyterian/Weill Cornell Medical Center, New York, New York; 2 Division of Gastroenterology and Hepatology, Department of Medicine, NewYork-Presbyterian/Weill Cornell Medical Center, New York, New York; 3 Department of Pathology and Laboratory Medicine, NewYork-Presbyterian/Weill Cornell, Medical Center, New York, New York

**Keywords:** IMHMV, Ischemic Colitis, Preoperative Diagnosis, Mesenteric Ischemia, Liver Transplant, Endoscopy

## Abstract

Idiopathic myointimal hyperplasia of the mesenteric veins is a rare cause of ischemic colitis resulting from luminal obliteration of medium- to large-sized mesenteric veins, which may mimic inflammatory bowel disease and infectious colitis, typically diagnosed following surgical resection. Delays in diagnosis are common and often preceded by extended hospitalizations and failed medical therapies including antibiotics and anti-inflammatories. Here, we present a rare instance of the preoperative diagnosis of idiopathic myointimal hyperplasia of the mesenteric veins in a woman who had previously received a liver transplant.

## Introduction

Idiopathic myointimal hyperplasia of the mesenteric veins (IMHMV) is a rare nonthrombotic cause of ischemic colitis, defined by luminal obliteration of medium- to large-sized mesenteric veins, often clinically mimicking inflammatory bowel disease or ischemic colitis.^1^ First described in 1991, it most often involves the rectosigmoid colon of younger to middle-aged males.^2^^,^^3^ Symptoms are generally nonspecific and chronic, although abdominal pain/diarrhea may be acute onset.^2^ Generally, the diagnosis is based on histologic sections of the vessels obtained from the resected specimen. Diagnostic delays leading to prolonged hospitalizations and failed treatments are often observed. We present a rare case of preoperative histologic diagnosis of IMHMV in a post–liver transplant patient.

## Case Report

A 41-year-old woman with a history including gastroesophageal reflux disease, former smoking (7.5 pack-years), provoked pulmonary embolism, severe alcohol use disorder (in remission for 2 years), and alcohol-associated cirrhosis status post uncomplicated deceased donor liver transplant 10 months prior presented with diffuse abdominal pain and distension, anorexia, nausea, and progressive nonbloody diarrhea (up to 25 stools/d) while on amoxicillin-clavulanate for recently diagnosed acute mastitis. Home medications include acamprosate, aspirin (81 mg), escitalopram, gabapentin, levetiracetam, pantoprazole, galcanezumab, rimegepant, and tacrolimus.

**Figure 1:**
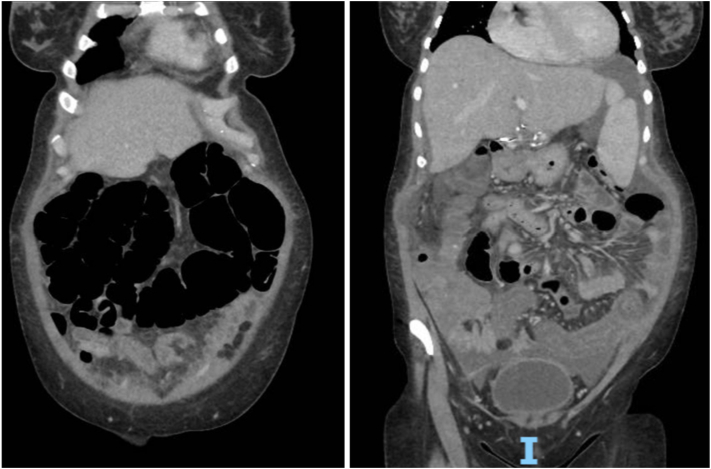
CT images demonstrating severe left-sided colitis, distension of the transverse colon, and fluid-filled distension of the cecum, up to 6 cm, without evidence of obstruction, toxic megacolon, or bowel perforation, and small to moderate volume ascites. CT, computed tomography.

**Figure 2:**
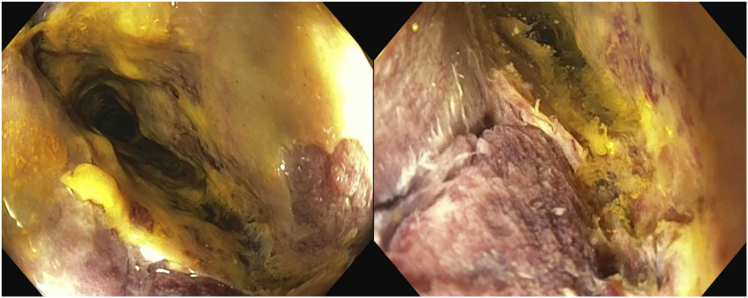
Outside hospital–performed flexible sigmoidoscopy images showing diffuse severe inflammation characterized by pseudomembranes, mucous, deep ulcerations, and stricture formation.

Initial workup revealed *Clostridioides difficile* stool test polymerase chain reaction positive, however toxin assay negative. Serial computed tomography imaging demonstrated progressive mural thickening/edema and pericolonic inflammatory stranding of middescending colon to rectum, air-filled distension of the transverse colon, fluid-filled distension of the cecum, and small to moderate ascites (Figure 1). She received empiric oral vancomycin for 5 days, switched to oral fidaxomicin on day 7, with intravenous metronidazole added on day 10. Flexible sigmoidoscopy (day 12) showed diffuse severe inflammation/deep mucosal ulcerations in the rectosigmoid colon, pseudomembranes, and a nontraversable sigmoid stricture (Figure 2). Biopsies revealed necroinflammatory debris without viable mucosal tissue for examination. She was transferred to our hospital on day 14.

On arrival, physical examination revealed sinus tachycardia, diffuse abdominal distension/tenderness, and rebound tenderness/guarding. Jaundice, edema, and asterixis were absent. Laboratory test results revealed hemoglobin 8 g/dL (baseline 14 g/dL), C-reactive protein 12 mg/L, and albumin 2.4 g/dL, with negative stool studies.

**Figure 3:**
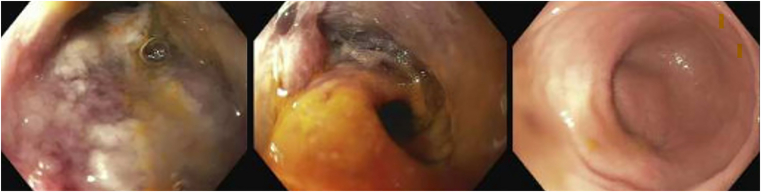
Endoscopic images showing sigmoid colon with severe congestion, erythema, ischemia, and pseudomembranes (left), stenosis at 30 cm (middle), and rectal sparing (right).

**Figure 4:**
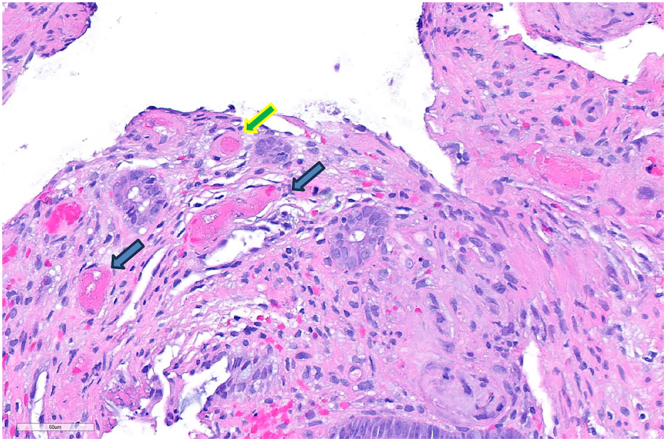
Sigmoid biopsy specimen revealing subendothelial hyalinization (blue arrows) and fibrin thrombus (green arrow) consistent with IMHMV.

Flexible sigmoidoscopy #2 (day 15) was notable for sigmoid pseudomembranes, severe congestion, erythema, ischemia, and stricture (nontraversable past 30 cm), with rectal sparing (Figure 3). Fecal microbiota transplantation was performed. Biopsies revealed regenerative colonic mucosa, fibropurulent exudates, prominent dilated lamina propria capillaries, and fibrin thrombi, raising suspicion for *C difficile* infection vs ischemic colitis. She received fidaxomicin, intravenous metronidazole, and rectal vancomycin postendoscopy, without improvement. Flexible sigmoidoscopy #3 (day 20) showed progressive congestive sigmoid colopathy and stricture (nontraversable past 20 cm). That evening, she developed worsened abdominal pain/distension. Computed tomography showed unchanged findings. Paracentesis was negative for bacterial peritonitis. Multidisciplinary discussions were held; however, operative intervention was deferred in the setting of ascites and malnutrition. She subsequently underwent colonoscopy and fecal microbiota transplantation #2 (day 25). The sigmoid stricture was traversed endoscopically, revealing diseased mucosa to 50 cm, normal-appearing transverse colon/hepatic flexure, and mild ascending colon inflammation. Sigmoidoscopy #3 biopsies resulted, revealing colonic pseudomembranes, ischemia/focal mucosal necrosis, subendothelial hyalinization, and fibrin thrombi consistent with IMHMV (Figure 4). The diagnosis was confirmed via colonoscopic biopsies from day 25.

**Figure 5:**
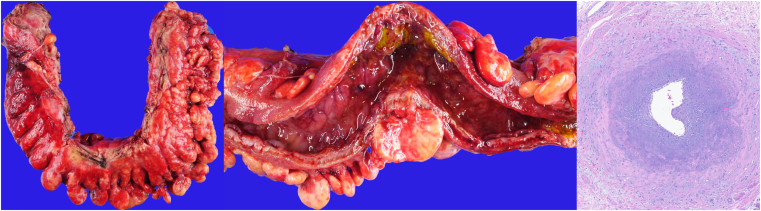
Gross surgical specimen revealing thick pericolic fat thickening (“wrapping”) (left) and mucosal thickening, erythema, ulceration, and pseudomembranes (middle); histology showing myointimal hyperplasia affecting a large mesenteric vessel (right).

**Figure 6:**
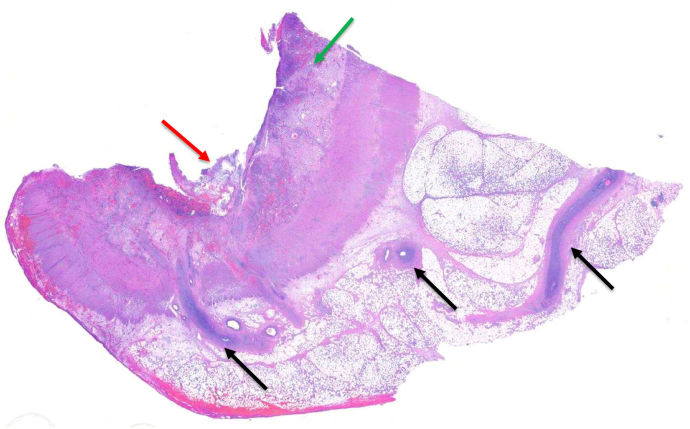
Surgical specimen histology revealing ulceration (red arrow), submucosal edema (green arrow), and venous obliteration by myointimal hyperplasia (black arrows).

She underwent repeat paracentesis (day 29), revealing secondary peritonitis, treated with 7 days of piperacillin-tazobactam. Parenteral nutrition was initiated for surgical optimization. She underwent laparoscopic-converted-to-open left hemicolectomy with end colostomy/Hartmann pouch (day 44). The surgical specimen revealed contained colonic perforation, pericolic fat thickening, mucosal thickening, erythema, ulceration, and pseudomembranes (Figure 5), and histology revealed mesenteric venous luminal obliteration (Figures 5 and 6). She improved postoperatively and was discharged on day 53 (postoperative day 9). At 3 weeks postdischarge, she reported resolving abdominal distension/discomfort and near-normal oral intake.

## Discussion

To the best of our knowledge, this is the second instance of preoperative IMHMV diagnosis made in a post–liver transplant patient.^4^ This rare, poorly-understood cause of colonic ischemia is defined by the classic histologic features of arteriolized capillaries, subendothelial fibrin deposits, and perivascular hyalinization.^5^ It has been hypothesized that IMHMV may result from chronic mechanical stress on mesenteric veins, as may occur with colonic hypermobility and intermittent volvulus, leading to tension and myointimal hyperplasia of the large-caliber veins.^5^^,^^6^ It is possible our patient’s prior abdominal surgery increased her risk for such mechanical bowel stress. Although tacrolimus is associated with endothelial injury and drug-induced thrombotic microangiopathy, this does not match the injury patterns typical of IMHMV, which itself is defined by the hallmarks of venous involvement and absence of thrombosis.

The sigmoid colon is most often affected (91%).^2^ The most common endoscopic findings—erythema/inflammation (92%), edema (85%), and ulceration (74%)—were present in our patient.^2^ Surgical resection is curative; however, delays in recognition/diagnosis occur in nearly one-third of patients.^2^ The median time to diagnosis is 4 months, and as demonstrated here, multiple endoscopies are frequently performed prior to diagnosis.^2^ In our case, *C difficile* colonization confounded the diagnosis of IMHMV. Early preoperative diagnosis of IMHMV may reduce unnecessary therapies, minimize complications, and decrease time to surgical management. As demonstrated here and emphasized in prior research, multidisciplinary discussions/close collaboration between specialists is crucial to minimizing diagnostic delay and facilitating treatment.^7^, ^8^, ^9^ Currently, no formal guidelines exist to help navigate discussions or decisions related to safety/optimization for surgery. Ascites and malnutrition impacted our patient’s surgical candidacy, delaying definitive treatment. Multidisciplinary discussions for IMHMV patients are instrumental in navigating the complexity of this rare disease, and balancing patient advocacy with safety in these cases can be especially challenging.
